# Supplementary material for: Bilateral interactions of optic-flow sensitive neurons coordinate course control in flies
Source: Nat Commun. 2024 Oct 12;15:8830. doi: 10.1038/s41467-024-53173-w (PMC11470938; doi:10.1038/s41467-024-53173-w)
Supplement: Supplementary file 1 — Supplementary Information [file 41467_2024_53173_MOESM1_ESM.pdf]

Supplementary Figures

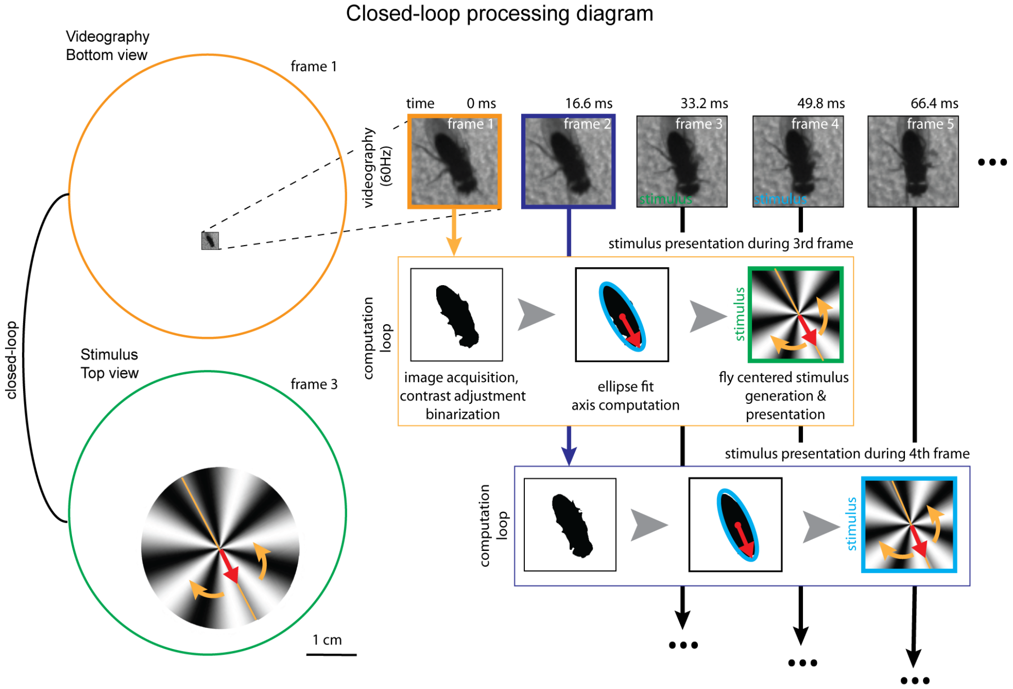

Supplementary Figure 1. Closed-loop paradigm. Fly behavior was sampled at 60 Hz. In parallel, the contour of the fly was extracted after pixel intensity thresholding, followed by an ellipse fit to this contour. The centroid and the angle of the major axis of the fitted ellipse were used to update the stimulus such that the pinwheel was centered on the body of the fly and the midline of the pinwheel was aligned with the body axis. This computation was performed in <5 ms. However, there was a total delay of < 50 ms (3 frames) for stimulus rendering. Here we show an example of 5 consecutive frames when the fly was in the midst of a fast turn, showing that even with this delay, the closed-loop paradigm was able to track the fly and update the visual stimulus effectively.

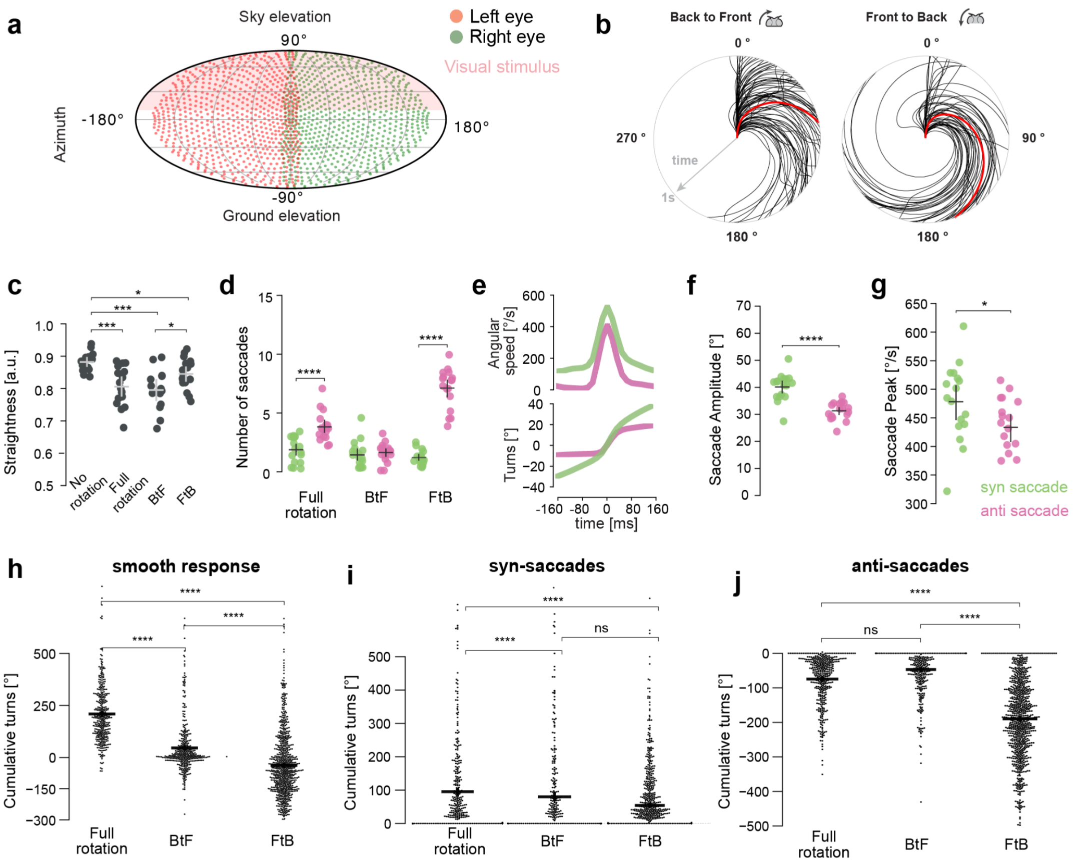

Supplementary Figure 2. Path straightness and properties of saccades for CantonS flies. a. Fly's visual field of view (from[38]) with the superimposed coverage of the visual stimuli used for behavioral experiments, presented as a Mollweide 2D projection. Circles represent viewing directions of ommatidia **b.** Polarplots showing the change in orientation for FtB and BtF immediately after stimulus onset during the first second (time increases radially). Black, single trials, red average. Note - in contrast to all other plots, we plot the difference with respect to the position of the stimulus, not the direction of motion. For clarity, we have removed trials where the fly did not respond during the first second and trials where the animal moved in the opposite direction. **c.** Path straightness of CantonS flies in response to full-field, BtF and FtB rotation. **d.** Number of syn- and anti-saccades per trial for CantonS flies. **e.** Mean angular speed (top) and turns (bottom) during a syn-saccade and an anti-saccade. **f.** Total turns made per saccade (in a ~160 ms window centered on the saccade peak) for syn- and anti-saccades by CantonS flies. **g.** Maximum angular speed during a saccade for syn- and anti-saccades for CantonS flies. **h.** Smooth cumulative angular displacement. Each dot represents one trial, data is pooled for all flies. Bars: mean ± SEM **i.** Same as (h), for syn-saccadic responses **j.** Same as h, for anti-saccadic responses. Mann-Whitney U test was applied in all panels, $*p < 0.05$, $**p < 0.01$, $***p < 0.001$, $****p < 0.0001$. Number of flies: n = 17. Exact *p*-values for each experiment are listed in Suppl. Data 1. Source data are provided as a Source Data file.

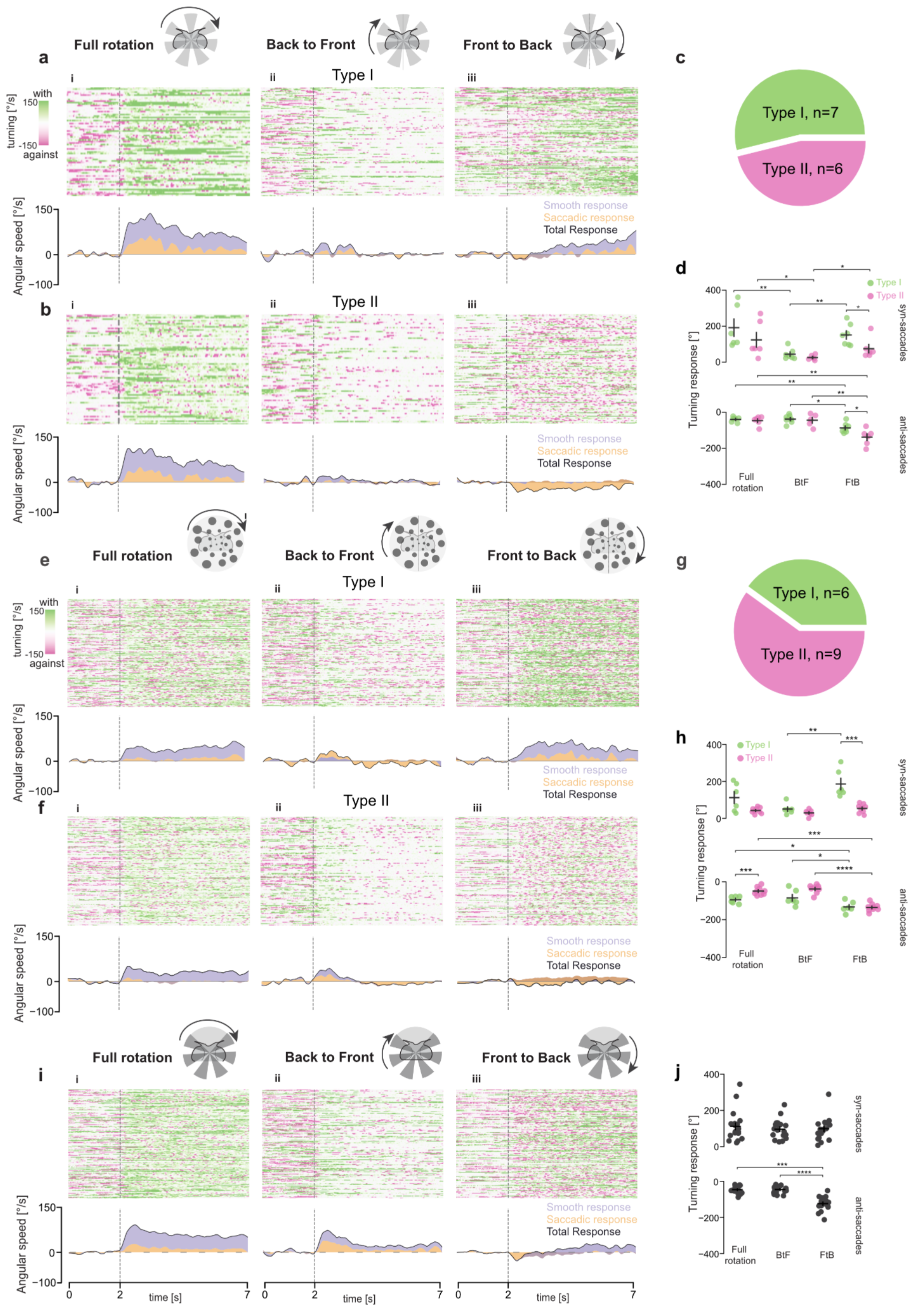

Supplementary Figure 3. The anti-saccadic response is stimulus parameter dependent. a,b. Turning response of CantonS flies to (i) full-field, (ii) unilateral Back to Front (BtF) and (iii) unilateral Front to Back (FtB) rotation stimulus with 25% contrast pinwheel. Top, Angular speed raster shows randomly selected trials. Each row corresponds to one trial, each column to one frame (~16 ms). Bottom, Stacked plot showing mean angular speed and the contribution of smooth and saccadic turning**.** Flies were assigned type 1 or 2 based on K-means clustering of the average angular speed for each fly with n_clusters=2. **c.** proportion of flies belonging to Type1 and Type2 **d.** Mean of syn-saccadic (top) and anti-saccadic (bottom) cumulative angular displacement per trial. **e,f.** same as in (a,b) for random dots stimulus **g.** Same as (c) for random dots stimulus **h.** Same as (d) for random dots stimulus **i.** Same as (a) for motion outside the region of binocular overlap (grey region in the schematic, 40°). **j.** Same as (d) for motion outside binocular overlap. Bars: mean ± SEM. Mann-Whitney U test was applied in all panels, $*p < 0.05$, $**p < 0.01$, $***p < 0.001$, $****p < 0.0001$. Number of flies: for 25% contrast (a-d): n=13, for starfield stimulus (e-g): n=15, for stimulus outside binocular overlap:n=19. Exact *p*-values for each experiment are listed in Suppl. Data 1. Source data are provided as a Source Data file. Schematic drawings credited to Laura Burnett.

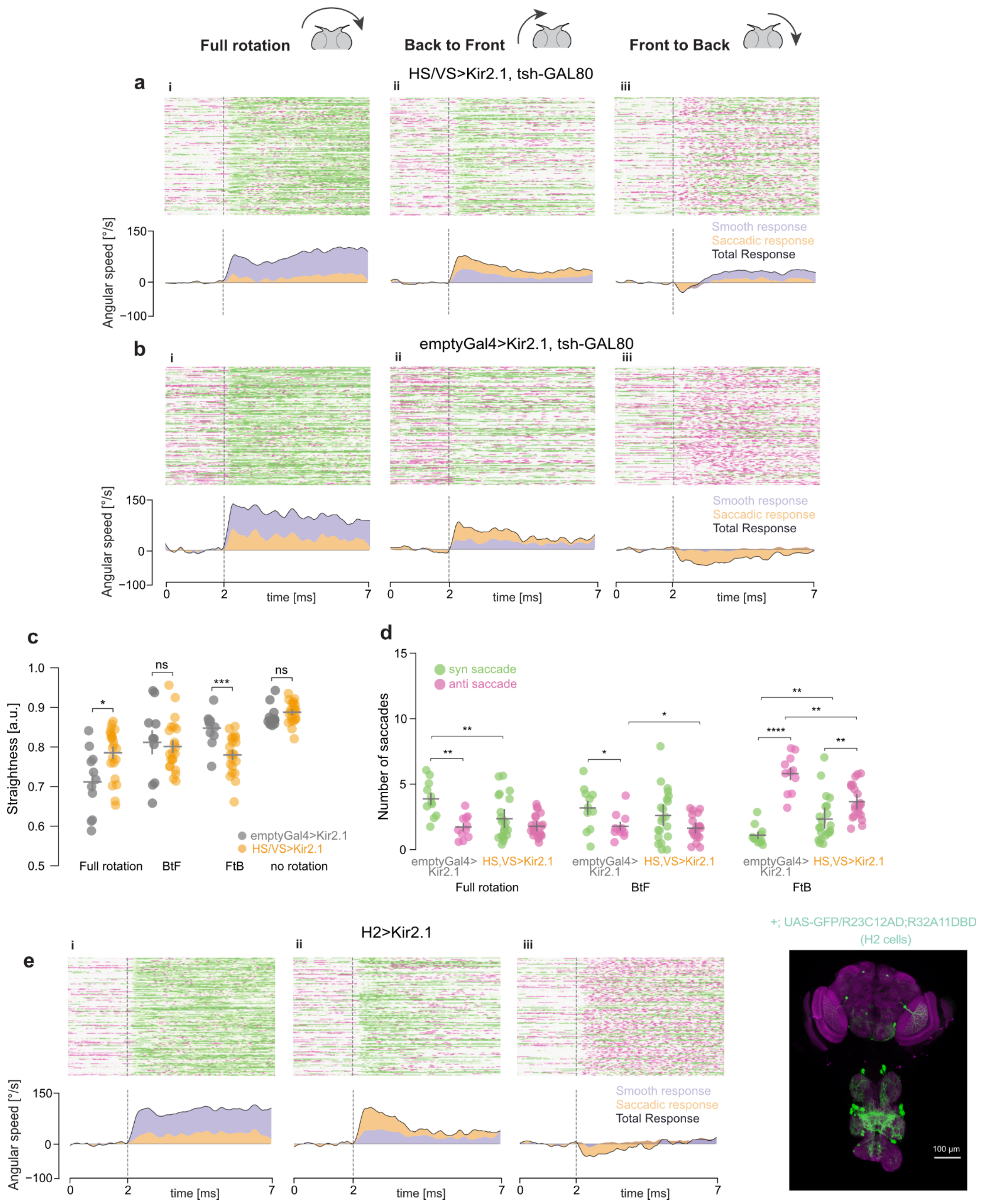

Supplementary Figure 4. Path straightness and properties of saccades for UAS-Kir2.1 control and HS & VS > Kir2.1 flies. a. Turning response of emptyGal4>Kir2.1, tshGal80 flies to (i) full-field, (ii) unilateral Back to Front (BtF) and (iii) unilateral Front to Back (FtB) rotation stimulus with 25% contrast pinwheel. Top, Angular speed raster shows randomly selected trials. Each row corresponds to one trial, each column to one frame (~16 ms). Bottom, Stacked plot showing mean angular speed and the contribution of smooth and saccadic turning**.** b.same as (a) for HS,VS>Kir2.1, tshGal80 flies **c.** Path straightness for Flp-control and Flp-shakB flies in response to full-field, BtF and FtB rotation. **d.** Number of syn- and anti-saccades per trial for Flp-control and Flp-shakB flies. **e.** right, same as (a) for H2>Kir2.1, tshGal80 flies. left, maximum z-projection of Kir2.1::EGFP expression by the R23C12AD,R32A11DBD-splitGAL4 line, used to silence H2 cells. Mann-Whitney U test was applied in all panels, $*p < 0.05$, $**p < 0.01$, $***p < 0.001$, $****p < 0.0001$. Number of flies: HS,VS>Kir2.1,tsh-Gal80 = 21, emptyGal4>Kir2.1,tsh-Gal80 = 11. Exact *p*-values for each experiment are listed in Suppl. Data 1. Source data are provided as a Source Data file. Schematic drawings credited to Laura Burnett.

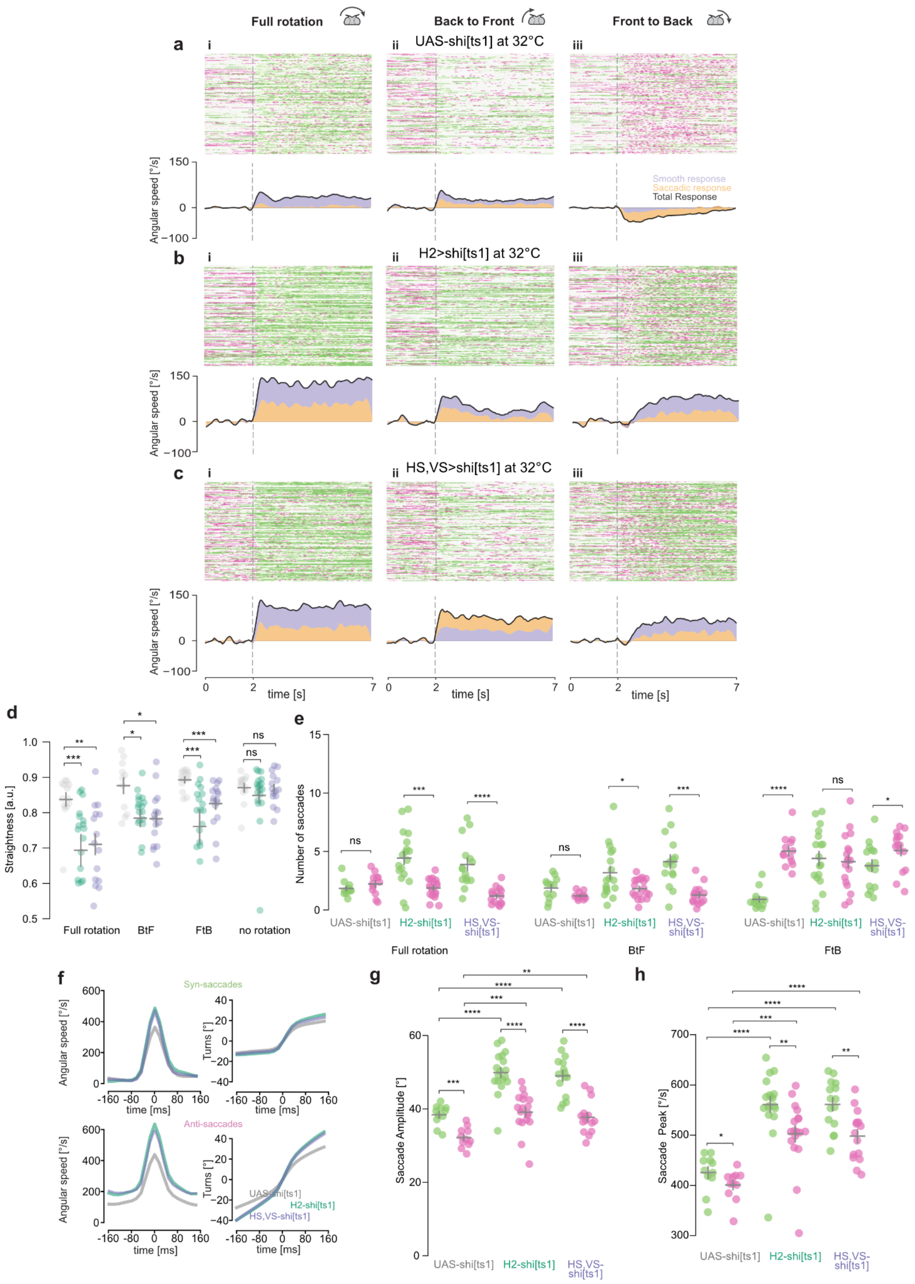

Supplementary Figure. 5 Summary of Shibire Experiments a. Turning response of UAS-shi[ts1] control flies at 32°C to (i) full-field, (ii) unilateral Back to Front (BtF) and (iii) unilateral Front to Back (FtB) rotation stimulus. Top, Angular speed raster shows randomly selected trials. Each row corresponds to one trial, each column to one frame (~16 ms). Bottom, Stacked plot showing mean angular speed and the contribution of smooth and saccadic turning**. b.** Same as (a) for H2>shi[ts1] at 32°C **c.** Same as (a) for HS,VS>shi[ts1] at 32°C **d.** Path straightness for Flp-control and Flp-shakB flies in response to full-field, BtF and FtB rotation. **e.** Number of syn- and anti-saccades per trial for Flp-control and Flp-shakB flies. **f.** Mean angular speed (left) and turns (right) during a syn-saccade and an anti-saccade. **g.** Total turns made per saccade (in a 160 ms window centered on the saccade peak) for syn- and anti-saccades in Flp-control and Flp-shakB flies. **h.** Maximum angular speed during a saccade for syn- and anti-saccades in Flp-control and Flp-shakB flies. Mann-Whitney U test was applied in all panels, $*p < 0.05$, $**p < 0.01$, $***p < 0.001$, $****p < 0.0001$. Number of flies: HS,VS>shi[ts1] = 16, H2>shi[ts1] = 18, UAS-shi[ts1] = 13. Exact p-values for each experiment are listed in Suppl. Data 1. Source data are provided as a Source Data file. Schematic drawings credited to Laura Burnett.

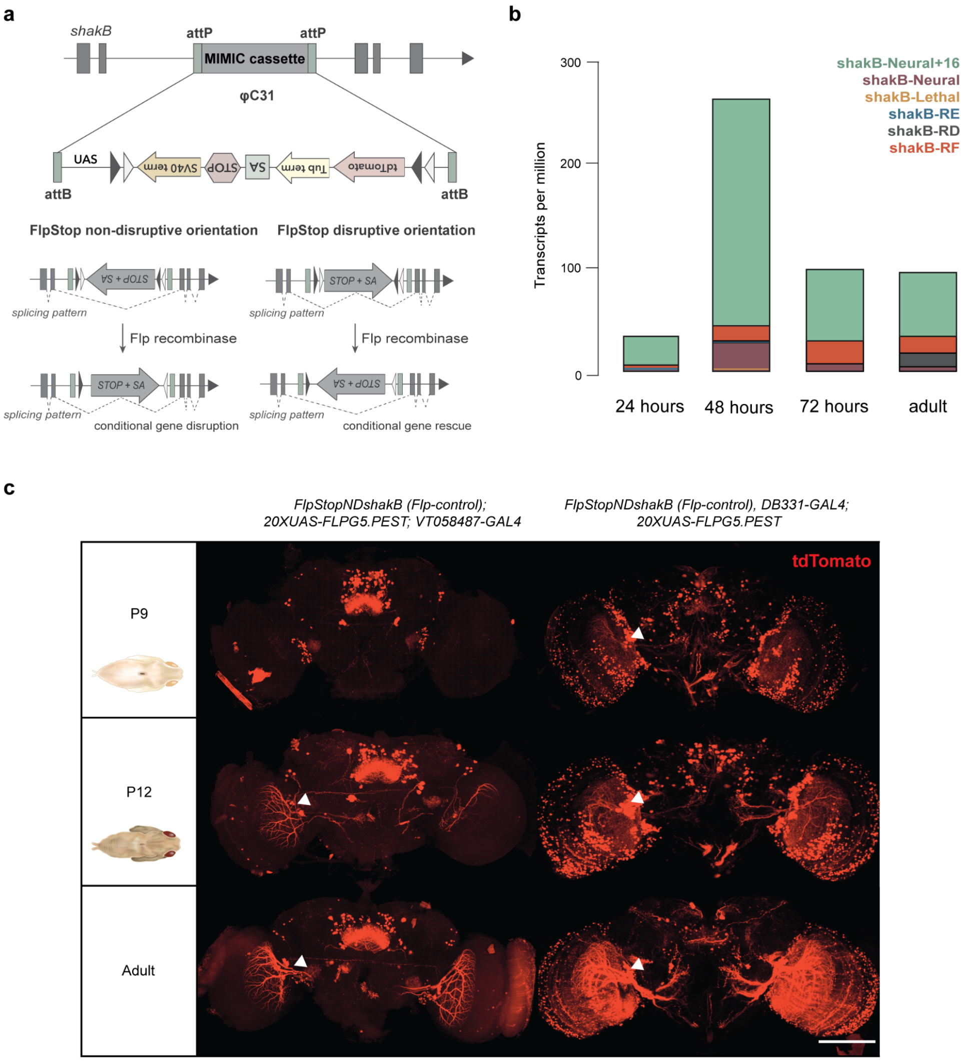

Supplementary Figure 6. FlpStop technique for disruption of the *shakB* gene. a. Schematic of the structure of the FlpStop cassette and of the gene disruption mechanisms[41]. The cassette is integrated into MiMIC insertion using PhiC31-integrase. The FlpStop cassette contains a splice acceptor (SA), transcriptional terminators (Tub α 1 terminator and the SV40), and stop codons in three frames (STOP). The cassette can be integrated into disrupting (FlpStopD) or non-disrupting (FlpStopND) orientation. The disrupting orientation can be used for a constitutive gene knock-out, while the non-disrupting orientation can trigger cell-specific gene inactivation in combination with flippase (Flp) and a GAL4-driver line. **b.** The expression of shakB isoforms in the optic lobe of *Drosophila* throughout pupal development. The analysis was performed on single-cell sequencing data of transcriptomes in the developing *Drosophila* visual system (see Methods). Isoform shakB-Neural+16 comprises shakB-RH, shakB-RI, shakB-RG; shakB-Neural corresponds to shakB-RC, and shakB-Lethal to shakB-RA. **c.** The dynamics of the inversion of the FlpStop cassette using two distinct LPTC-specific driver lines - DB331-Gal4 and VT058487-Gal4. The expression of the tdTomato was used as a marker of the cassette inversion. The onset of the cassette inversion is around P9 for DB331-Gal4 and around P12 for VT058487-Gal4, somas of LPTCs are indicated with white arrows. Scale bar: 100 µm. Source data are provided as a Source Data file.

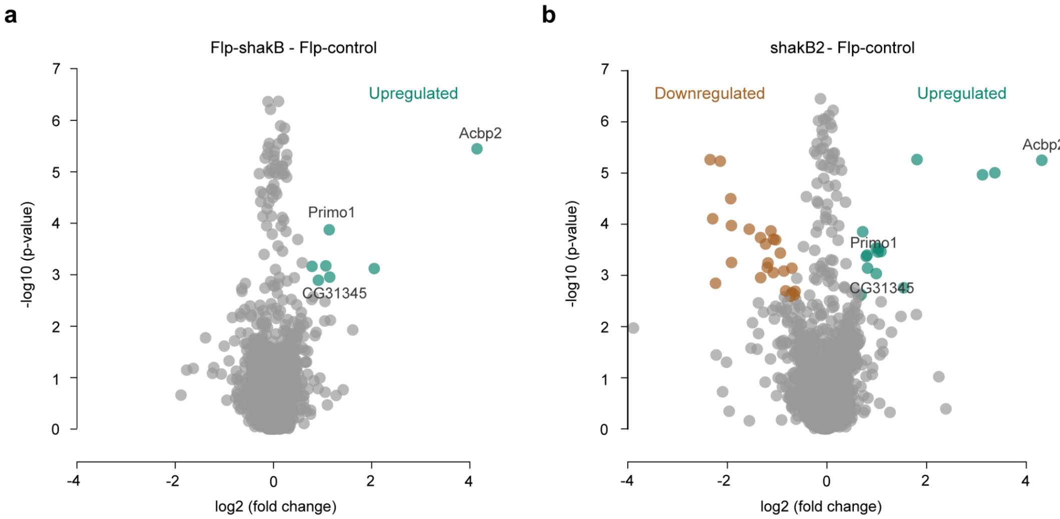

Supplementary Figure 7. Proteomic analysis of Flp-shakB and *shakB*^{2} mutant brains. Total protein lysates obtained from fly brains were analyzed through liquid chromatography mass-spectrometry (LC-MS). **a.** Protein level quantification (fold change) and statistical significance assessment (p-value) for Flp-shakB mutant model were performed against Flp-control control flies. Proteins that showed significant changes in expression levels are depicted in brown (down-regulated) and green (up-regulated). **b.** The same for the *shakB2* mutant model. Source data are provided as a Source Data file.

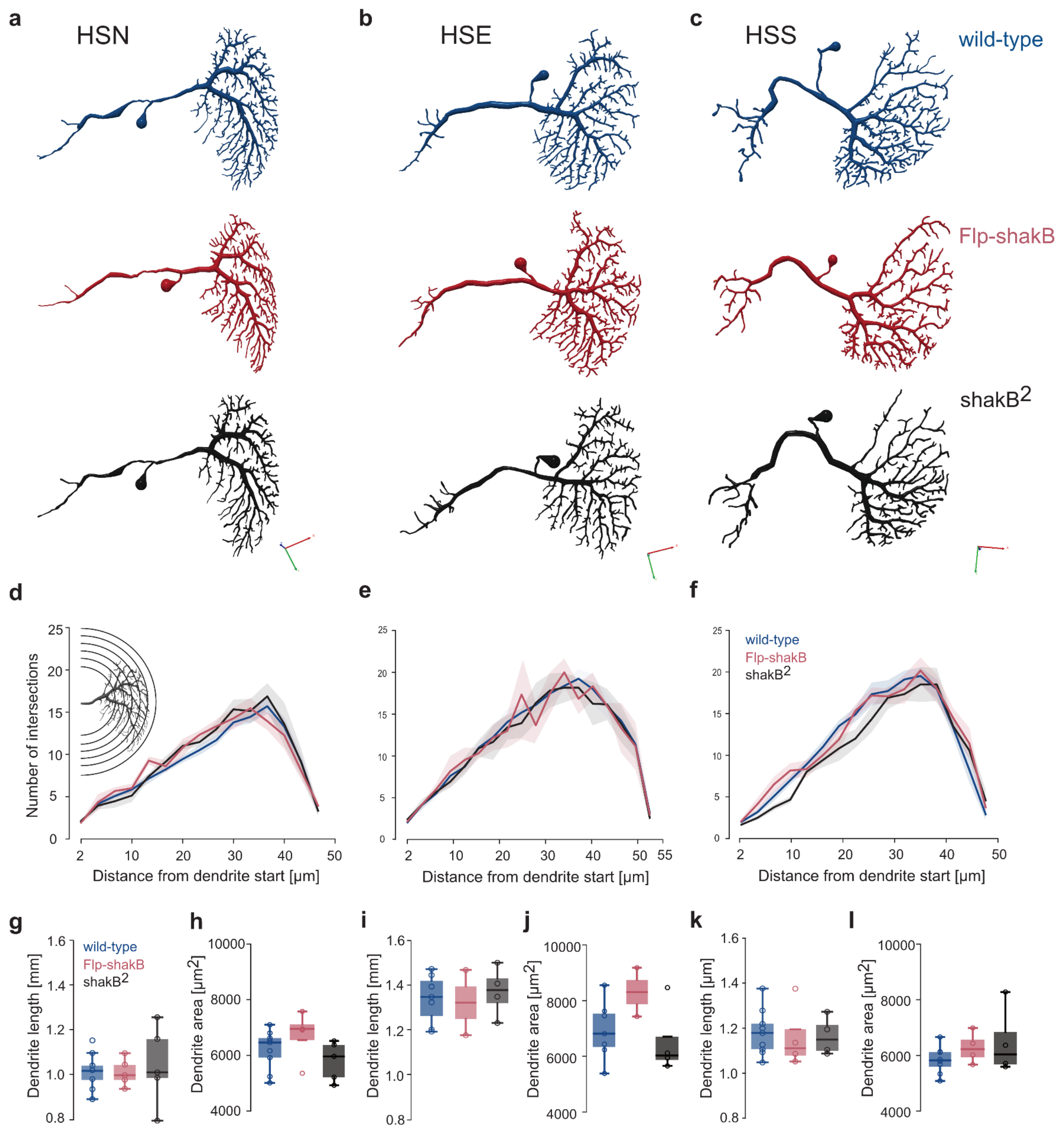

Supplementary Figure 8. Loss of gap junctions does not affect the morphology of dendrites in HS cells. a-c. Examples of reconstructed HSN, HSE and HSS cells for every genotype. d-f. Sholl intersection profile of HS dendrites in the wild type and two mutant lines (mean ± SEM). The number of intersections for each HS type was equalized between genotypes. g,i,k. Total length of HSN, HSE and HSS dendrites in the wild type and two mutant lines. h, j, l. Dendritic field area for HSN, HSE and HSS in the wild type and two mutant lines. Upper/lower limit and inner horizontal lines of the box plots represent upper/lower quartile and median, respectively; whiskers indicate 1.5 interquartile range from upper/lower quartiles. Source data are provided as a Source Data file.

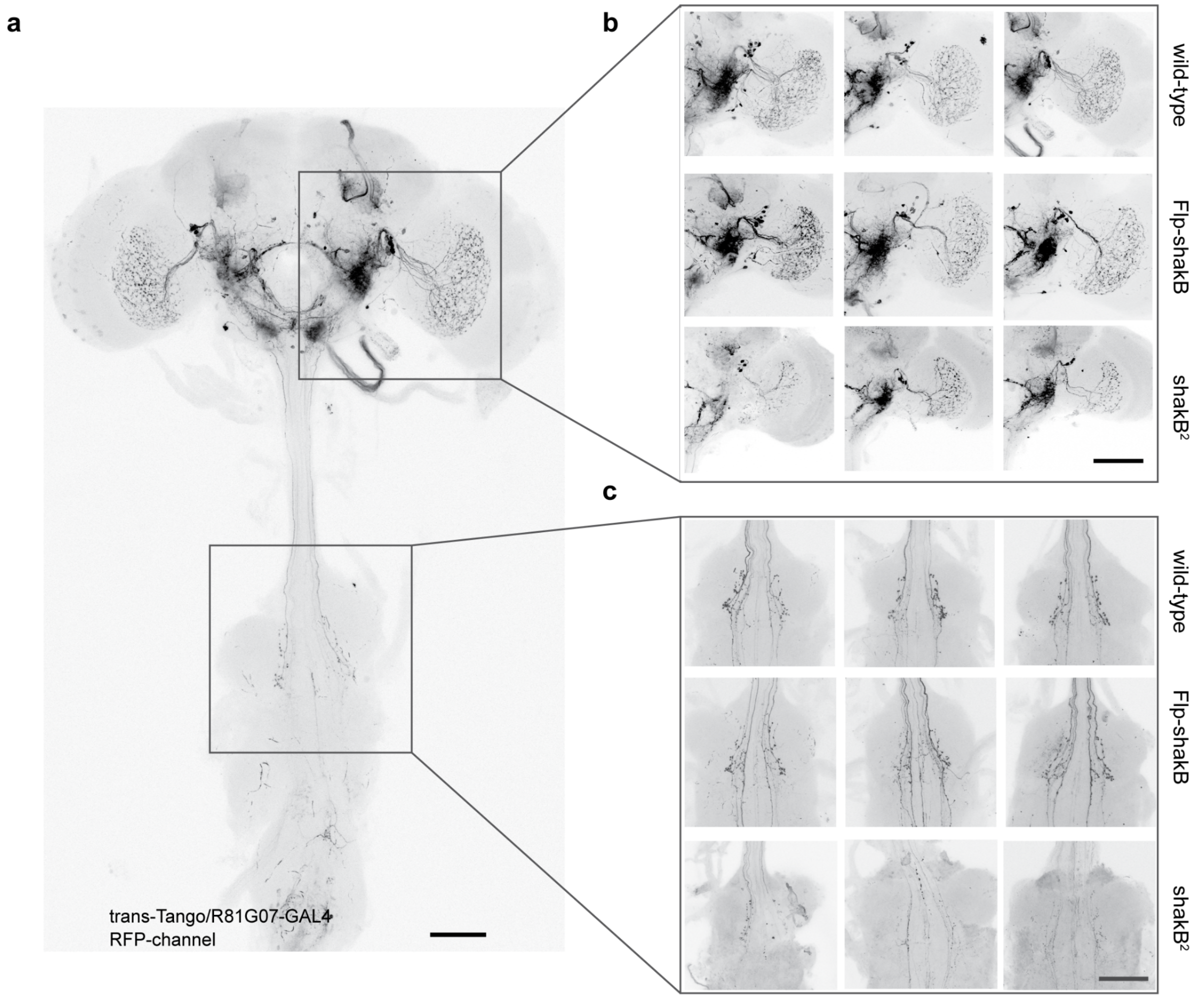

Supplementary Figure 9. Trans-synaptic labeling does not reveal the loss of chemical postsynaptic partners of HS cells in Flp-shakB flies. a. Expression pattern of the postsynaptic marker (tdTomato) in HS>trans-Tango flies. b. Examples of labeled synaptic partners of HS cells in the optic lobe and the posterior slope in the wild type and two mutant lines. c. Examples of labelled synaptic partners of HS cells in the VNC in the wild type and two mutant lines. Scale bar: 50 µm.

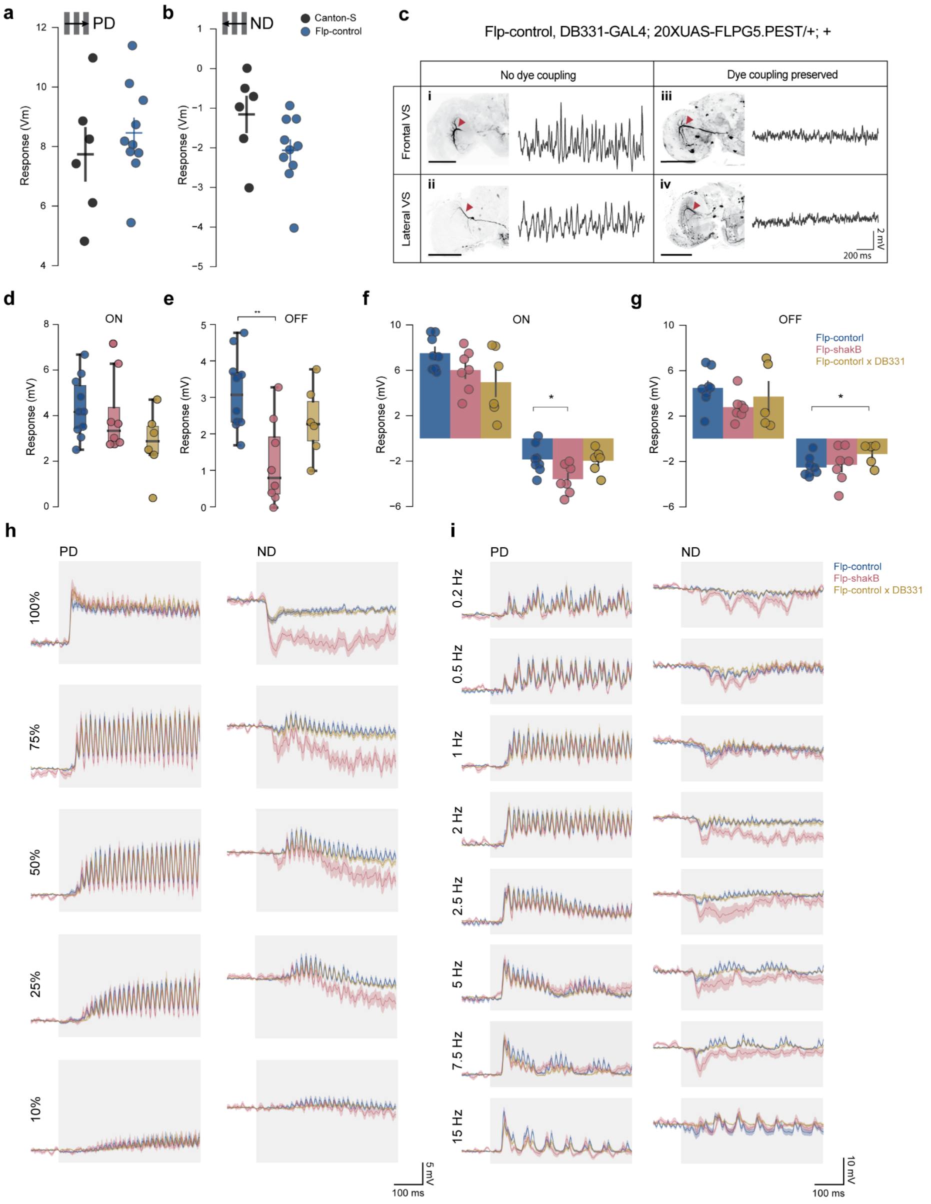

Supplementary Figure 10. **Additional characterization of FlpStop-induced shakB inactivation on physiological properties of LPTCs.** a. Average voltage changes of HS cells in Canton-S and Flp-control flies during 2 s presentation of square-wave gratings moving in PD (mean ± SEM). b. Same as in (a) for null direction (ND). c. Fast membrane oscillations of LPTCs are cell-intrinsic. Example traces of membrane potential and neurobiotin coupling of VS cells in flies with LPTC-specific inactivation of ShakB protein. In contrast to VS cells preserving neurobiotin coupling (iii, iv), VS cells lacking neurobiotin coupling with other LPTC cells (i, ii) exhibit fast membrane fluctuations. Scale bar: 50 µm, red triangle indicates injected/recorded cell. d. Voltage response of HS cells in wild type and mutant flies to full-field light ON flash during 50 ms onset of the stimulus. e. Same as (d) for light OFF flash stimulus. Upper/lower limit and inner horizontal lines of the box plots represent upper/lower quartile and median, respectively; whiskers indicate 1.5 interquartile range from upper/lower quartiles. f. Average voltage responses of HS cells in wild-type and mutant flies to drifting ON-edges moving in PD and ND (mean± SEM). g. Same as (f) for OFF-edge. h. Average voltage response traces of HS cells in wild type and mutant flies to gratings with different contrast moving in PD and ND at a temporal frequency of 1 Hz (mean± SEM). Oscillations are a stimulus artefact due to the projector's refresh rate. i. Average voltage response traces of HS cells in wild type and mutant flies to gratings moving with different temporal frequencies in PD and ND (mean± SEM). For a,b,d-g Mann-Whitney U test was applied, $*p < 0.05$, $**p < 0.01$. Source data are provided as a Source Data file.

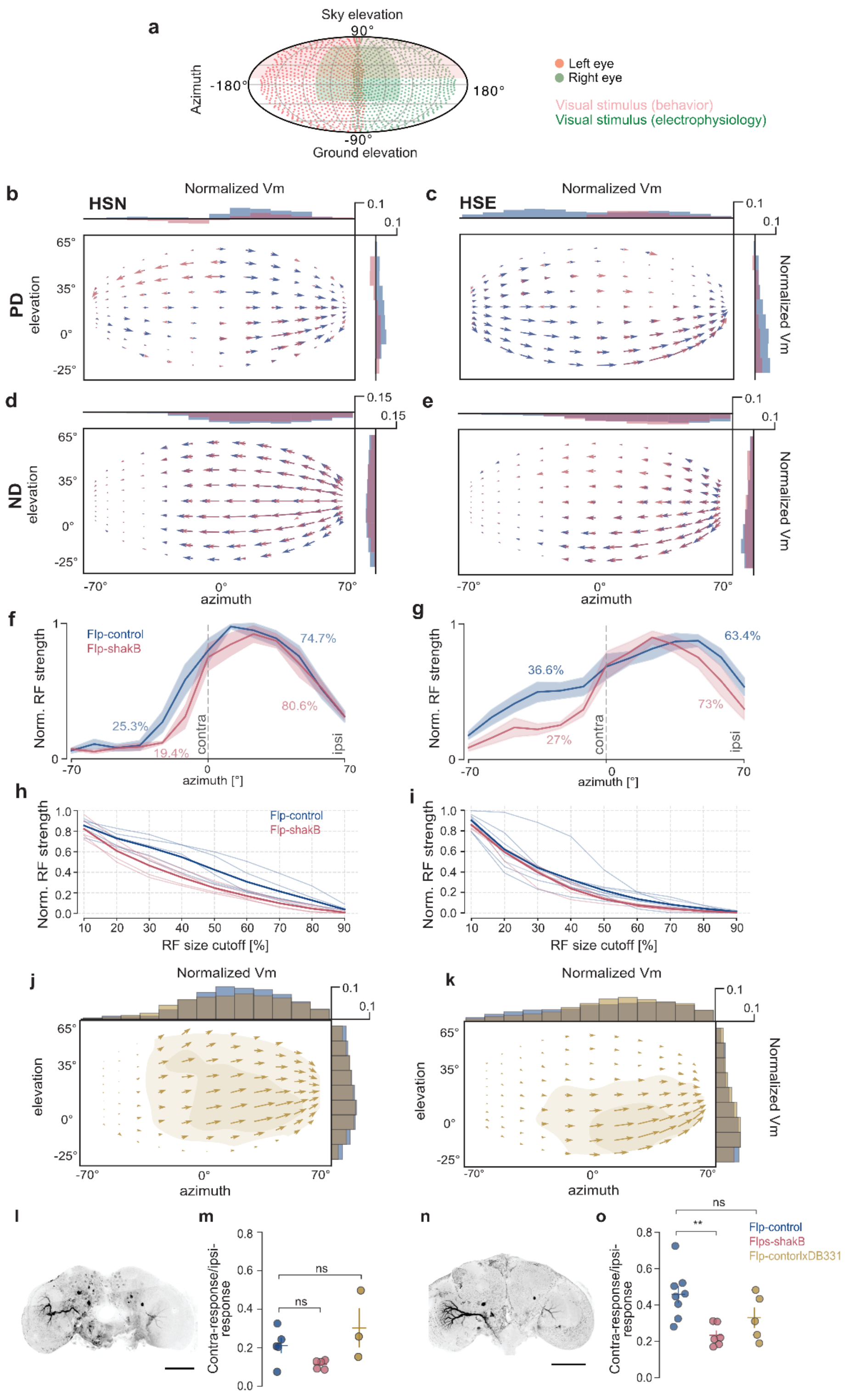

Supplementary Figure 11. Additional characterization of FlpStop-induced *shakB* inactivation on the receptive fields (RF) of HS cells. a. Fly's visual field of view (from[38]) with the superimposed coverage of the visual stimuli used for behavioral and electrophysiological experiments, presented as a Mollweide 2D projection. Circles represent viewing directions of ommatidia **b,c.** The receptive fields of (**b**) HSN and (**c**) HSE cells for Flp-control and Flp-shakB flies to the local preferred direction (LPD) were calculated by only considering the horizontal movement in the PD of the cells. Horizontal and vertical bar plots show average response along azimuth and elevation, respectively. **d,e.** Same as (**b,c**) for local motion in ND. **f.** Normalized strength of responses of HSN neurons across the azimuth, computed as the sum of all local motion sensitive responses at a given azimuth. **g.** Same as (**f**) for HSE

cells. **h.** Size of RF of HSN cells (as a proportion of total recorded visual field) for different cutoffs of the maximal response. **i.** Same as (**h**) for HSE neurons. **j.** Spatial RF reconstructed from the responses to local motion stimulus of HSN cells in flies with induced inversion of FlpStop-cassette in LPTCs (*Flp-control, DB331-GAL4; UAS-Flp/+;+*). Light-shaded and dark-shaded areas represent 30% and 60% of the maximal strength of the response. Horizontal and vertical bar plots show response along azimuth and elevation, respectively, for Flp-control and induced mutant flies. **k.** Same as (**j**), for HSE cells. **l.** An example of neurobiotin injection into an individual HSN cell in *Flp-control, DB331-GAL4; UAS-Flp* flies. Scale bar 100 µm. **m.** Relative strength between the contralateral and ipsilateral visual fields of individual HSN cells. Bars: mean ± SEM. **n.** Same as (**l**) for an HSE neuron. **n.** Same as (**l**) for HSE neurons. Mann-Whitney U test was applied in (**m**) and (**o**), $*p < 0.05$, $**p < 0.01$. Number of cells recorded: (**b**), (**d**), (**f**), (**h**): Flp-control = 5, Flp-shakB = 5; (**c**), (**e**), (**g**), (**i**): Flp-control= 8, Flp-shakB = 6; (**j**): n=3; (**k**): n=5. Exact *p*-values for each experiment are listed in Suppl. Data 1. Source data are provided as a Source Data file.

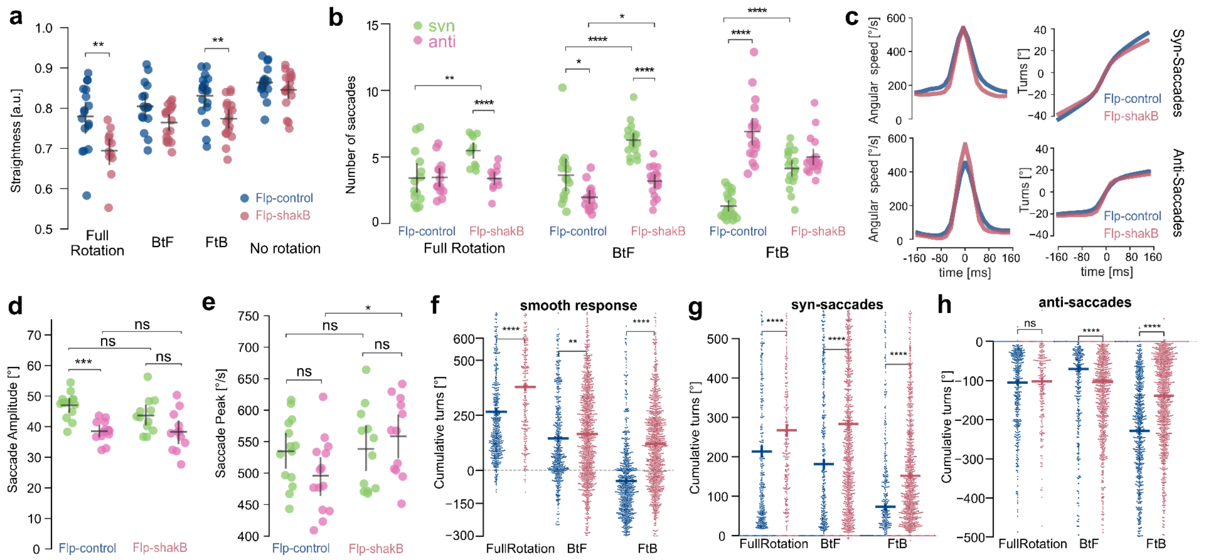

Supplementary Figure 12. Path straightness and properties of saccades for Flp-control and Flp-shakB flies. a. Path straightness for Flp-control and Flp-shakB flies in response to full-field, BtF and FtB rotation. **b.** Number of syn- and anti-saccades per trial for Flp-control and Flp-shakB flies. **c.** Mean angular speed (left) and turns (right) during a syn-saccade and an anti-saccade. **d.** Total turns made per saccade (in a 160 ms window centered on the saccade peak) for syn- and anti-saccades in Flp-control and Flp-shakB flies. **e.** Maximum angular speed during a saccade for syn- and anti-saccades in Flp-control and Flp-shakB flies. **f.** Smooth cumulative angular displacement. Each dot represents one trial, data is pooled for all flies. Bars: mean ± SEM **g.** Same as in f, for syn-saccadic responses **h.** Same as in f, for anti-saccadic responses Mann-Whitney U test was applied in all panels, $*p < 0.05$, $**p < 0.01$, $***p < 0.001$, $****p < 0.0001$. Number of flies: Flp-control = 13, Flp-shakB = 11. Exact *p*-values for each experiment are listed in Suppl. Data 1. Source data are provided as a Source Data file.

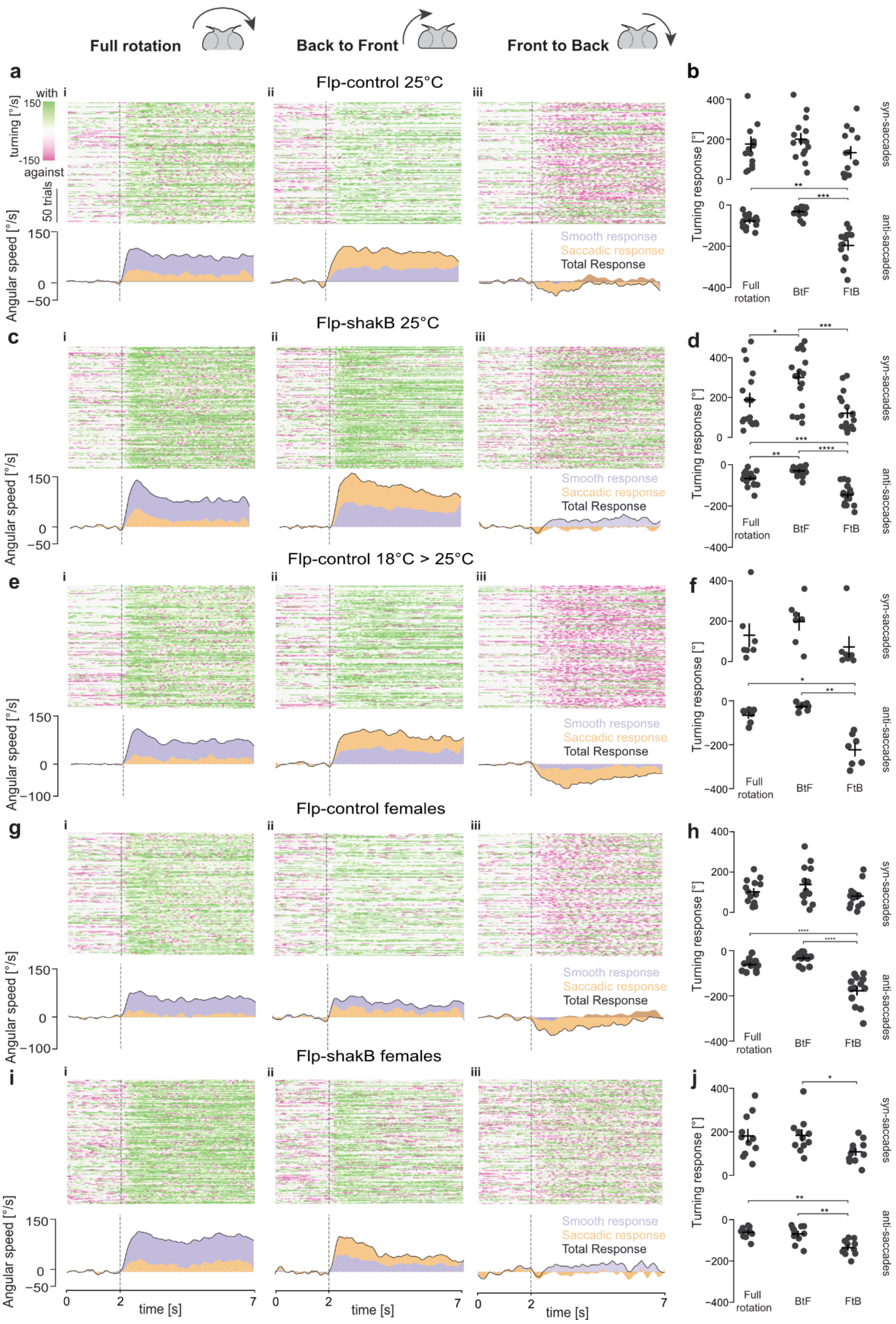

Supplementary Figure 13. The anti-saccadic response is not sex-specific, but its strength can be modulated by rearing conditions. a. Turning response of Flp-control flies reared at 25°C to (i) full-field, (ii) unilateral Back to Front (BtF) and (iii) unilateral Front to Back (FtB) rotation stimulus. Angular speed raster shows randomly selected trials. Each row corresponds to one trial, each column to one frame (~16 ms). **b.** Mean of syn-saccadic (top) and anti-saccadic (bottom) cumulative angular displacement per trial. Bars: mean ± SEM. **c.** same as in (a) for Flp-shakB flies reared at 25°C. **d.** Same as in (**b**) for Flp-shakB flies reared at 25°C. **e.** Same as in (**a**) for Flp-control flies reared at 18°C through larval and pupal stages and transferred to 25°C after the eclosion. **f.** Same as in (**b**) for Flp-control flies reared at 18°C through larval and pupal stages and transferred to 25°C after the eclosion. **g.** Same as in (**a**) for Flp-control female flies. **h.** Same as in (**b**) for Flp-control female flies. **i.** Same as in (**a**) for Flp-shakB female flies. **j.** Same as in (**b**) for Flp-shakB female flies. Number of flies: Flp-control at 25°C:

n = 15, Flp-shakB at 25°C: n = 18, Flp-control at 25°C transferred to 18°C: n = 11, Flp = control female: n = 11, Flp-shakB female: n = 11. Exact *p*-values for each experiment are listed in Suppl. Data 1. Source data are provided as a Source Data file. Schematic drawings credited to Laura Burnett.

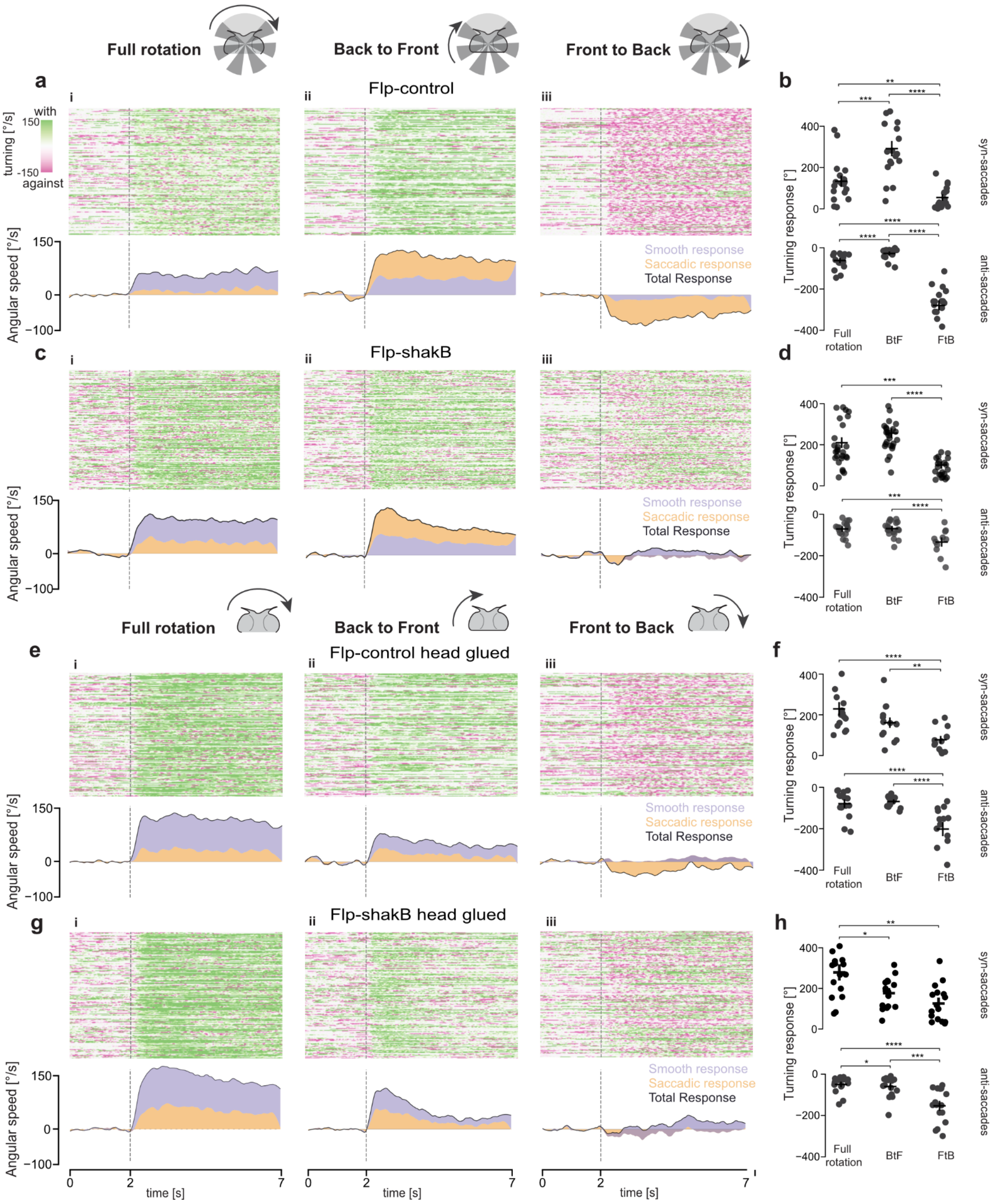

Supplementary Figure 14. The anti-saccadic response is not dependent on the binocular field of view nor on head movements. a. Turning response of Flp-control flies to (i) full-field, (ii) unilateral Back to Front (BtF) and (iii) unilateral Front to Back (FtB) rotation stimulus outside the region of binocular overlap (grey region in the schematic, 40°). Angular speed raster shows randomly selected trials. Each row corresponds to one trial, each column to one frame (~16 ms). **b.** Mean of syn-saccadic (top) and anti-saccadic (bottom) cumulative angular displacement per trial. Bars: mean ± SEM. **c.** Same as in (**a**) for Flp-shakB flies. **d.** Same as in (**b**) for Flp-shakB flies. **e-h.** Same as in (**a-d**) for flies with heads glued to the thorax to eliminate neck motion. Number of flies: Flp-control (for stimulus outside binocular overlap): n = 18, Flp-shakB(for stimulus outside binocular overlap): n = 15, Flp-control(glued head): n = 16, Flp-shakB(glued head): n = 16. Source data are provided as a Source Data file. Schematic drawings credited to Laura Burnett.

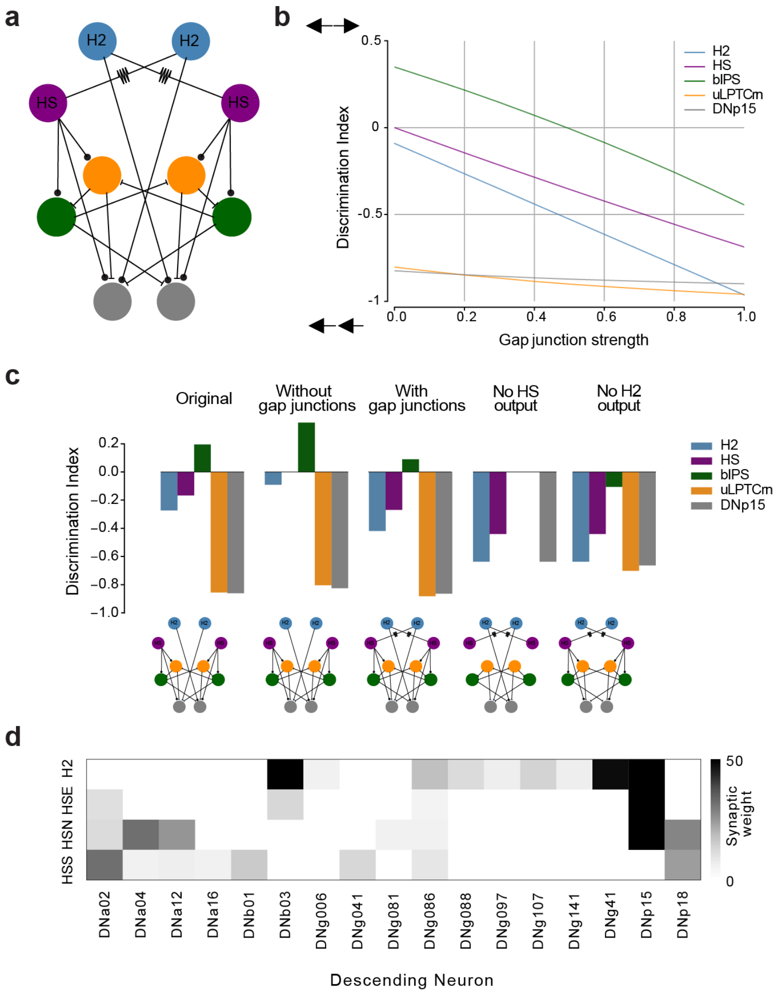

Supplementary Figure 15. Gap junctions enhance discrimination between translational and rotational stimuli. a. Schematic of a subset of the HS-H2 network (modified from [15]), each colour represents a different neuron type. **b**. Discrimination index (DI) between translational and rotational stimuli for each neuron type in the circuit on the left. A value of +1 indicates that the neuron responds differentially to forward and backward translation, while a value of -1 indicates that the neuron responds differentially to clockwise and counter-clockwise rotation. **c**. Comparison of DI for different configurations of the circuit for all neurons. Weights corresponding to specific connections in the model were changed to obtain the different configurations. The schematic at the bottom shows the state of the circuit after the weights were changed. **d**. Number of synapses made by HSN, HSE, HSS and H2 neurons onto descending neurons (DNs) based on data from the FlyWire connectome [71]. Only DNs where the number of synapses was greater than 5 were considered. Source data are provided as a Source Data file.
