# Supplementary material for: Bilateral interactions of optic-flow sensitive neurons coordinate course control in flies
Source: Nat Commun. 2024 Oct 12;15:8830. doi: 10.1038/s41467-024-53173-w (PMC11470938; doi:10.1038/s41467-024-53173-w)
Supplement: Supplementary file 3 — Description of Additional Supplementary Files [file 41467_2024_53173_MOESM3_ESM.pdf]

Description of Additional Supplementary Files

File name: Supplementary Data 1
Description: Detailed statistical analysis.

File name: Supplementary Movie 1
Description: Example of full-field and unilateral optic flow stimulation in wild-type CantonS flies.

File name: Supplementary Movie 2
Description: Example of full-field and unilateral optic flow stimulation in *HS,VS>Kir2.1* flies.

File name: Supplementary Movie 3
Description: Example of full-field and unilateral optic flow stimulation in *Flp-control* flies.

File name: Supplementary Movie 4
Description: Example of full-field and unilateral optic flow stimulation in *Flp-shakB* flies.
