# Supplementary material for: Bilateral interactions of optic-flow sensitive neurons coordinate course control in flies
Source: Nat Commun. 2024 Oct 12;15:8830. doi: 10.1038/s41467-024-53173-w (PMC11470938; doi:10.1038/s41467-024-53173-w)
Supplement: Supplementary file 9 — Reporting Summary [file 41467_2024_53173_MOESM9_ESM.pdf]

nature portfolio

Corresponding author(s): Maximilian Jösch

Last updated by author(s): Sep 4, 2024

Reporting Summary

Nature Portfolio wishes to improve the reproducibility of the work that we publish. This form provides structure for consistency and transparency in reporting. For further information on Nature Portfolio policies, see our Editorial Policies and the Editorial Policy Checklist.

Statistics

For all statistical analyses, confirm that the following items are present in the figure legend, table legend, main text, or Methods section.

n/a	Confirmed	
☐	☒	The exact sample size (*n*) for each experimental group/condition, given as a discrete number and unit of measurement
☐	☒	A statement on whether measurements were taken from distinct samples or whether the same sample was measured repeatedly
☐	☒	The statistical test(s) used AND whether they are one- or two-sided<br>*Only common tests should be described solely by name; describe more complex techniques in the Methods section.*
☒	☐	A description of all covariates tested
☐	☒	A description of any assumptions or corrections, such as tests of normality and adjustment for multiple comparisons
☐	☒	A full description of the statistical parameters including central tendency (e.g. means) or other basic estimates (e.g. regression coefficient) AND variation (e.g. standard deviation) or associated estimates of uncertainty (e.g. confidence intervals)
☐	☒	For null hypothesis testing, the test statistic (e.g. *F*, *t*, *r*) with confidence intervals, effect sizes, degrees of freedom and *P* value noted<br>*Give P values as exact values whenever suitable.*
☒	☐	For Bayesian analysis, information on the choice of priors and Markov chain Monte Carlo settings
☒	☐	For hierarchical and complex designs, identification of the appropriate level for tests and full reporting of outcomes
☒	☐	Estimates of effect sizes (e.g. Cohen's *d*, Pearson's *r*), indicating how they were calculated

Our web collection on statistics for biologists contains articles on many of the points above.

Software and code

Policy information about availability of computer code

Data collection	Behavioral data was collected using custom code written in Python.<br>Electrophysiological data was acquired using custom software written in LabView and stimuli were generated using custom code written in Matlab.<br>Microscopy images were processed in Fiji.<br>Neuronal morphology was reconstructed using Neutube and Imaris software (Imaris9.3.1)
Data analysis	All code is available on GitHub: https://github.com/joesch-lab/Bilateral-course-control

For manuscripts utilizing custom algorithms or software that are central to the research but not yet described in published literature, software must be made available to editors and reviewers. We strongly encourage code deposition in a community repository (e.g. GitHub). See the Nature Portfolio guidelines for submitting code & software for further information.

Data

Policy information about availability of data

All manuscripts must include a data availability statement. This statement should provide the following information, where applicable:

- Accession codes, unique identifiers, or web links for publicly available datasets
- A description of any restrictions on data availability
- For clinical datasets or third party data, please ensure that the statement adheres to our policy

Data used in the analysis uploaded to ISTA data repository : https://doi.org/10.15479/AT:ISTA:17488

Research involving human participants, their data, or biological material

Policy information about studies with human participants or human data. See also policy information about sex, gender (identity/presentation), and sexual orientation and race, ethnicity and racism.

Reporting on sex and gender	*Use the terms sex (biological attribute) and gender (shaped by social and cultural circumstances) carefully in order to avoid confusing both terms. Indicate if findings apply to only one sex or gender; describe whether sex and gender were considered in study design; whether sex and/or gender was determined based on self-reporting or assigned and methods used. Provide in the source data disaggregated sex and gender data, where this information has been collected, and if consent has been obtained for sharing of individual-level data; provide overall numbers in this Reporting Summary. Please state if this information has not been collected. Report sex- and gender-based analyses where performed, justify reasons for lack of sex- and gender-based analysis.*
Reporting on race, ethnicity, or other socially relevant groupings	*Please specify the socially constructed or socially relevant categorization variable(s) used in your manuscript and explain why they were used. Please note that such variables should not be used as proxies for other socially constructed/relevant variables (for example, race or ethnicity should not be used as a proxy for socioeconomic status). Provide clear definitions of the relevant terms used, how they were provided (by the participants/respondents, the researchers, or third parties), and the method(s) used to classify people into the different categories (e.g. self-report, census or administrative data, social media data, etc.) Please provide details about how you controlled for confounding variables in your analyses.*
Population characteristics	*Describe the covariate-relevant population characteristics of the human research participants (e.g. age, genotypic information, past and current diagnosis and treatment categories). If you filled out the behavioural & social sciences study design questions and have nothing to add here, write "See above."*
Recruitment	*Describe how participants were recruited. Outline any potential self-selection bias or other biases that may be present and how these are likely to impact results.*
Ethics oversight	*Identify the organization(s) that approved the study protocol.*

Note that full information on the approval of the study protocol must also be provided in the manuscript.

Field-specific reporting

Please select the one below that is the best fit for your research. If you are not sure, read the appropriate sections before making your selection.

☒ Life sciences ☐ Behavioural & social sciences ☐ Ecological, evolutionary & environmental sciences

For a reference copy of the document with all sections, see nature.com/documents/nr-reporting-summary-flat.pdf

Life sciences study design

All studies must disclose on these points even when the disclosure is negative.

Sample size	No calculation was performed to predetermine sample sizes. The number of flies was sufficient to perform non-parametric testings, for all experimental and control groups, unless mentioned otherwise in Methods
Data exclusions	No flies were excluded. However, in the analysis of behavioral data, all trials in which the animal showed no motion during the entirety of the trial (both pre-stimulus and post-stimulus period) were rejected from further analysis as described in Methods. For electrophysiological data, recordings of poor quality due to sub-optimal access were discarded from further analysis.
Replication	All experiments for all genotypes were done with multiple batches, that is, flies were taken from different crossings and the results were consistent across batches.
Randomization	Wherever different conditions (contrast, speed etc.) for stimuli were used in behavioral as well as electrophysiological experiments, the sequence of the appearance of conditions was randomized.

Blinding	Blinding was not possible for experiments since experimenters were aware of the genotype of the flies before testing.

Reporting for specific materials, systems and methods

We require information from authors about some types of materials, experimental systems and methods used in many studies. Here, indicate whether each material, system or method listed is relevant to your study. If you are not sure if a list item applies to your research, read the appropriate section before selecting a response.

Materials & experimental systems

n/a	Involved in the study	
☐	☒	Antibodies
☒	☐	Eukaryotic cell lines
☒	☐	Palaeontology and archaeology
☐	☒	Animals and other organisms
☒	☐	Clinical data
☒	☐	Dual use research of concern
☒	☐	Plants

Methods

n/a	Involved in the study	
☒	☐	ChIP-seq
☒	☐	Flow cytometry
☒	☐	MRI-based neuroimaging

Antibodies

Antibodies used	Antibodies and dilutions used in the immunohistochemistry experiments: anti-shakB rabbit serum antibody (kind gift of Alexander Borst, Max Planck Institute for Biological Intelligence, Martinsried, Germany; 1:800), goat anti-GFP (Abcam, ab6673; 1:500), goat anti-RFP (Rockland, 200-101-379S; 1:500), rabbit anti-GFP (Thermo Fisher, A11122; 1:500), donkey anti-goat AF488 (Abcam, ab150129; 1:1000), donkey anti-goat AF594 (Thermo Fisher, A32758; 1:1000), donkey anti-rabbit AF594 (Thermo Fisher, A21207; 1:1000), CF594 rabbit anti-RFP (Biotium, 20422; 1:500).<br>Antibodies and dilutions used in the western blot experiments: anti-ShakB (1:3000, Innovagen AB), IRDye 800CW goat anti-rabbit (1:15000, LI-COR Biosciences).
Validation	All antibodies, apart from the anti-ShakB serum that was generated for this study, have been previously validated. The affinity of anti-ShakB was compared between brain extracts of WT and ShakB KO mutant flies.

Animals and other research organisms

Policy information about studies involving animals; ARRIVE guidelines recommended for reporting animal research, and Sex and Gender in Research

Laboratory animals	For behavior: 3 to 6 days old male and female Drosophila melanogaster reared on a standard cornmeal-molasses agar medium at either 18 °C or 25 °C and 60 % humidity and kept on a 12 h light/12h dark cycle.<br>For electrophysiology : 1-day old males reared on a standard cornmeal-molasses agar medium at 18 °C and 60 % humidity and kept on a 12 h light/12h dark cycle.<br><br>Strains used in this study:<br>Canton S (wildtype) / BDSC<br>shakB[2]; +; + / Augustin Hrvoje<br>shakBFlpStopND, w+; +; + / this paper<br>shakBFlpStopD, w+; +; + / this paper<br>w1118; +; VT058487-GAL4 / Vienna Drosophila Resource Center (VDRC)<br>w1118; +; 10XUAS-IVS-eGFPKir2.1/TM6B / Eugenia Chiappe<br>DB331-GAL4; +; + / Alexander Borst<br>w+;tsh-GAL80/Cyo;VT058487-GAL4/+ / this paper<br>w+; R23C12-p65.AD; R32A11-GAL4.DBD / Eugenia Chiappe<br>w+; +; UAS-shi[ts1] / BDSC<br>y1, w*, UAS-myrGFP.QUAS-mtdTomato-3xHA; trans-Tango;+ / BDSC<br>w1118; +; R81G07-GAL4 / BDSC<br>w*;10XUAS-IVS-mCD8::GFP;+ / BDSC<br>w1118; 20XUAS-FLPG5.PEST; + / BDSC<br>w+; 20XUAS-SPARC2-I-mCD8::GFP/Cyo; + / BDSC<br>y1, w*; 20XUAS-IVS-PhiC31; + / Thomas Clandinin<br>w1118; VT058487-p65.AD; + / BDSC<br>w1118; +; VT000343-GAL4.DBD / BDSC
Wild animals	No wild animals were used in the study
Reporting on sex	Both male and female flies were used in the study. However, only male flies were used in the main figures to draw the primary conclusions due to reduced penetrance and high phenotypic variability in female mutant flies owing to the fact that shakB gene is on X-chromosome.

Field-collected samples	No field-collected animals were used in the study
Ethics oversight	No ethical approval or guidance was required for experiments with Drosophila

Note that full information on the approval of the study protocol must also be provided in the manuscript.
